# Chronic environmental stress enhances tolerance to seasonal gradual warming in marine mussels

**DOI:** 10.1371/journal.pone.0174359

**Published:** 2017-03-23

**Authors:** Ionan Marigómez, Maria Múgica, Urtzi Izagirre, Inna M. Sokolova

**Affiliations:** 1 CBET Research Group, Research Centre for Experimental Marine Biology and Biotechnology (PiE-UPV/EHU), Areatza, Plentzia-Bizkaia, Basque Country, Spain; 2 Marine Biology, Institute for Biosciences, University of Rostock, Rostock, Germany; Gettysburg College, UNITED STATES

## Abstract

In global climate change scenarios, seawater warming acts in concert with multiple stress sources, which may enhance the susceptibility of marine biota to thermal stress. Here, the responsiveness to seasonal gradual warming was investigated in temperate mussels from a chronically stressed population in comparison with a healthy one. Stressed and healthy mussels were subjected to gradual temperature elevation for 8 days (1°C per day; fall: 16–24°C, winter: 12–20°C, summer: 20–28°C) and kept at elevated temperature for 3 weeks. Healthy mussels experienced thermal stress and entered the time-limited survival period in the fall, became acclimated in winter and exhibited sublethal damage in summer. In stressed mussels, thermal stress and subsequent health deterioration were elicited in the fall but no transition into the critical period of time-limited survival was observed. Stressed mussels did not become acclimated to 20°C in winter, when they experienced low-to-moderate thermal stress, and did not experience sublethal damage at 28°C in summer, showing instead signs of metabolic rate depression. Overall, although the thermal threshold was lowered in chronically stressed mussels, they exhibited enhanced tolerance to seasonal gradual warming, especially in summer. These results challenge current assumptions on the susceptibility of marine biota to the interactive effects of seawater warming and pollution.

## Introduction

With the advent of global climate change, rise in seawater surface temperature can severely affect coastal ecosystems [1,2]. Temperature elevation affects metabolic regulation in marine organisms and may induce thermal stress [3,4], which leads to reduced aerobic scope, depressed metabolism as well as disturbed physiological functions, reproduction and general health condition. As a result, less energy is allocated to growth, storage, defence and reproduction [4–6]. Furthermore, seawater warming acts in concert with contaminants and other stress sources operating in marine ecosystems [7,8].

Exposure to and susceptibility against pollutants may be strongly affected by seawater warming [7–9]. Temperature modifies pollutants' toxicity by altering their bioavailability, uptake and transformation; thus, high metabolic rates associated with elevated temperatures promote bioaccumulation and result in augmented toxicity [8,9]. Thermal stress may exaggerate the toxic effects of pollutants through increasing mitochondrial damage and oxidative stress, impairing physiological capacities and leading to energy imbalance [10]. Moreover, as aerobic scope declines at critically high temperatures, tolerance to pollutants may decline due to limitations in energy supply for detoxification and repair [11]. Pollutant exposure also influences the capacity of marine ectotherms to respond to thermal stress. Detrimental effects of pollutants on metabolism can result in energy-deficient conditions as the organism attempts to counteract temperature elevation [12] while coping with elevated basal metabolic demand due to the costs of up-regulated cellular protective mechanisms, which reduces aerobic scope, narrows thermal tolerance limits and shifts upper critical temperatures to lower values [8,9,11,13,14]. Consequently, marine ectotherms inhabiting polluted marine ecosystems may experience stronger effects of seawater warming.

In temperate coastal areas, mussels are dominant members of the rocky intertidal community and a major research target both for climate change and pollution studies [15–17]. In mussels, biological responses elicited by temperature elevation, pollution and other environmental stressors include oxidative stress [17–20], as well as impaired health condition (lysosomal responses [21–25] and histopathological alterations [25,26]), compromised reproduction [6,11] and disturbances of energy homeostasis [5,6,16]. Integrated metabolic response to temperature and pollution involves reorganizations at the molecular (i.e. activity of metabolic enzymes), cellular (i.e. lysosomal size, morphology and stability) and tissue levels (i.e. changes in the composition and integrity of digestive epithelia).

**Fig 1:**
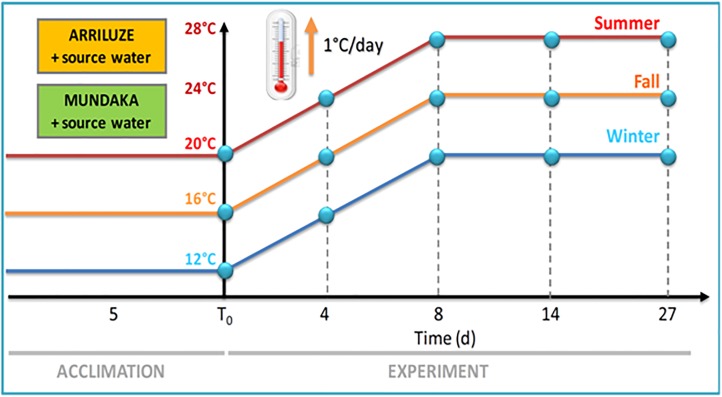
Experimental design diagram. Mussels, *M*. *galloprovincialis* were collected from Mundaka and Arriluze in fall, winter and summer and placed in tanks with their source seawater at 16, 12 and 20°C respectively for 5 days (acclimation). Then, temperature was gradually increased 1°C per day during 8 days (from 16°C to 24°C in fall, from 12°C to 20°C in winter from 20°C to 28°C in summer), and maintained at season temperature elevation until Day 27. Mussels (n = 5) were retrieved at days 0, 4, 8, 14 and 27 (after acclimation to elevated temperature; 20, 28 or 24°C).

We investigated the responses to seasonal gradual warming in temperate mussels, *Mytilus galloprovincialis*, from a mussel population chronically subject to multiple stress sources in the Bay of Biscay in comparison with a nearby healthy one. Upon collection, mussels were maintained under laboratory conditions in their corresponding source seawater, subjected to gradual temperature elevation for 8 days (1°C per day starting at the source temperature at each season: 16°C in fall, 12°C in winter, and 20°C in summer) and maintained at elevated temperature for additional 3 weeks (Fig 1). Molecular, cellular, tissue-level and energetic biomarkers, as well as gametogenesis were investigated at different time intervals.

## Materials and methods

Unless otherwise indicated, all chemicals and enzymes were purchased from Sigma- Aldrich (St. Louis, MO, USA), Roche (Indianapolis, IN, USA) or Fisher Scientific (Pittsburg, PA, USA) and were of analytical grade or higher.

### Characterization of the mussel populations

Mussels (*Mytilus galloprovincialis*) of 3.5–4.5 cm shell length (N = 780; 130 *per* estuary and season) were collected from 2 estuaries geographically close to each other (40 km) in the Basque Coast: Urdaibai and Bilbao. The Basque Government (Department of Fishing and Aquaculture) issued the permission for mussel collection at both locations, in compliance of the legal regulations in force for shellfish harvesting in public coastal domains in the Basque Country. No additional requirements were needed as they were invertebrates. Mundaka is a small village sited in the Urdaibai estuary (43°22′N, 2°40′W). Urdaibai was declared a Biosphere Reserve by United Nations Educational and Scientific Cultural Organization in 1984 and it comprises the estuary of the Oka River (Bay of Biscay). It is considered as one of the best preserved estuaries of the Northern Iberian Peninsula, with relatively low concentrations of organic pollutants and heavy metals [25,27–31]. Arriluze is a marina located at the right bank of the Bilbao estuary (43°20′N, 3°0′W); which has been subject to severe urban and industrial impact for decades and was still considered heavily polluted in the 1990s [25,27,29,31–34]. As a result of the increased captures and treatment of wastewater discharges, pollutant load to the Bilbao estuary was significantly reduced from 1993 to 2003, although moderate levels of PAHs and high levels of PCBs still persist [25,27–29,34]. We have chosen these two study sites because we have long-term data and archived specimens from these two areas since late 1980s [17,24–29,31,34] providing a wealth of environmental, physiological and ecological information on these populations and allowing us to make stronger inferences about the potential mechanisms and ecological consequences of the observed physiological changes.

Assessment of ecosystem health disturbance was conducted to confirm that each locality was characterised by a different level of environmental stress. For this purpose, mussels (N = 30 *per* season) from both localities were used as sentinels and the biological responses described below were used to construct an integrative biomarker index (Ecosystem Health Condition Chart: EHCC) in order to establish the following ecosystem health conditions [17]: “good ecosystem health condition”; “tolerable ecosystem health condition”; “delicate ecosystem health condition”; and “bad ecosystem health condition”. As a whole, Mundaka presented a ‘good ecosystem health condition’ whilst Arriluze ranged from ‘delicate ecosystem health condition’ in winter and fall to ‘bad ecosystem health condition’ in summer (Table 1).

**Table 1:** Summary of seasonal T_0_ values for studied biomarkers in each locality. LP (min); V*v*_L_ (μm^3^/μm^3^); S*v*_L_ (μm^2^/μm^3^); S/V_L_ (μm^2^/μm^3^); N*v*_L_ (1/μm^3^); V*v*_NL_ (μm^3^/μm^3^); V*v*_BAS_ (μm^3^/μm^3^); MET (μm); MLR (μm); MLR/MET (μm/μm); CTD ratio; TAOC (Teq μmol); PK (U/g prot); PEPCK (U/g prot); COX (U/g prot); HK (U/g prot); GP (U/g prot); GI; PSSF (%); and POA (%). Background levels: LP>20 [23–25,35,49–51]; V*v*_L(fall,summer)_<0.002 and V*v*_L(spring)_<0.0005 [22–25,35,50,51]; S*v*_L_<0.009 [22–25,35,50,51]; S/V_L_>4 [22–25,35,50,51]; N*v*_L(winter)_<0.004 and N*v*_L(summer)_<0.002 [22–25,35,50,51]; V*v*_NL(spring, summer)_<0.005 and V*v*_NL(fall)_<0.1 [51,52]; V*v*_BAS_<0.12 [26,35,51]; MLR/MET_(fall, summer)_<1.2 and MLR/MET_(spring)_<0.6 [26,35,51]. Different letters (a, b, c) indicate significant differences between seasons in mussels from each locality. Characters in bold indicate significant differences between localities for a given season after either Man Whitney’s *U*-test or Student’s *t*-test (*p*<0.05) (or χ^2^ for PSSF and POA). UDL: Under detection limit; PSSF: prevalence (%) of small sized mature follicles; POA: prevalence (%) of oocyte atresia.

	Healthy mussel population (Mundaka)			Stressed mussel population (Arriluze)		
	Fall	Winter	Summer	Fall	Winter	Summer
**LP**	**21.83±2.7**	18.12±1.9	21.46±2.6	**5.15±0.3**^**a**^	13.75±1.2^b^	**9.69±1.1**^**b**^
**Vv**_**L**_	**0.0036±0.0009**^**a**^	**0.0004±0.0002**^**b**^	**0.0003±0.0001**^**b**^	**0.0065±0.0020**	**0.0057±0.0030**	**0.0059±0.0020**
**Sv**_**L**_	**0.0105±0.002**^**a**^	**0.0026±0.001**^**b**^	**0.0025±0.001**^**b**^	**0.0152±0.003**	**0.0168±0.006**	**0.0152±0.002**
**S/V**_**L**_	3.01±0.40^a^	6.84±0.84^b^	**8.01±0.83**^**c**^	2.46±0.45	3.10±0.72	**2.68±0.37**
**Nv**_**L**_	0.0017±0.0003	0.0017±0.0006	0.0021±0.0007	0.0015±0.0002^a^	0.0028±0.0002^b^	0.0021±0.0002^c^
**Vv**_**NL**_	0.083±0.015	UDL	UDL	0.082±0.024^a^	0.025±0.012^b^	0.160±0.046^c^
**Vv**_**BAS**_	0.134±0.01	0.104±0.02	**0.114±0.02**	0.098±0.01^a^	0.101±0.04^a^	**0.188±0.03**^**b**^
**MET**	**13.10±0.6**	**13.60±1.6**	12.70±1.4	**14.17±0.7**	**15.00±2.9**	13.60±2
**MLR**	11.6±0.88	12.3±1.87	12.72±1.54	7.91±0.35^a^	8.21±1.04^a^	11.93±1.42^b^
**MLR/MET**	**0.92±0.09**	**0.93±0.20**	1.04±0.20	**0.58±0.03**^**a**^	**0.59±0.20**^**a**^	0.92±0.20^b^
**CTD ratio**	**0.20±0.40**^**a**^	**0.18±0.02**^**a**^	0.24±0.05	**0.68±0.13**^**a**^	**0.29±0.04**^**b**^	0.23±0.03^b^
**TAOC**	**20.83±3.00**^**a**^	**16.05±1.00**^**b**^	22.32±1.70^a^	**11.03±1.85**^**a**^	**11.39±0.65**^**a**^	18.63±0.71^b^
**PK**	37.14±7.3^a^	**111.43±17.9**^**b**^	53.39±4.1^a^	41.4±4.2	**35.61±1.1**	43.23±6.2
**PEPCK**	**8.87±2.0**^**a**^	31.68±4.5^b^	16.51±3.9^a^	**16.19±2**	27.13±5	22.12±5.3
**PK/PEPCK**	3.87±1.2^a^	**3.63±0.4**^**a,b**^	3.11±0.4^b^	2.74±0.5	**1.76±0.3**	2.73±0.9
**COX**	25.62±6.4^a^	59.47±12.9^b^	**78.13±18.1**^**b**^	32.86±9.9	41.93±7.6	**37.55±7.7**
**HK**	22.89±8.1	51.94±18.2	**36.19±5.5**	34.26±7.7^a^	65.27±6.7^b^	**19.71±4.0**^**a**^
**GP**	**16.07±2.8**^**a**^	11.35±2.5^a,b^	**5.29±1.8**^**b**^	**6.00±1.3**^**a**^	10.15±3.7^a,b^	**17.13±3.9**^**b**^
**GI**	**1.78±0.35**^**c**^	3.73±0.27^a^	2.87±0.24^b^	**2.93±0.33**^**a**^	4±0.1^b^	3.31±0.33^a,b^
**PSSF**	100^b^	0^c^	0^c^	58.33^b^	6.67^c^	**53.84**^**a,c**^
**POA**	0	30	10	0	20	0

### Mussel collection and experimental design

Mussels were collected in fall (November), winter (early March) and summer (July) 2009–2010 and transported to the laboratory within 1 h of collection. Mussels (N = 100 *per* experimental group) were placed in 45 L flow-through tanks with aerated seawater which was carried from the corresponding source localities to the laboratory. A different seawater experimental temperature was established for each season (16°C, 12°C and 20°C in fall, winter and summer, respectively) depending of the temperature at the time of collection (surface seawater temperature data at Bilbao station (43.40°N 3.13°W, 53 m depth) were obtained from www.puertos.es >Oceanografía y Meteorología >Redes de Medida>Red Costera; access dates: July 2009-April 2010). Food was provided as cultured live cells of algae *Isochrysis* spp. (20000 cells/mL, 4 μm size) by constant administration (1 L/day). Mussels were allowed to acclimate to laboratory conditions for 5 days. No mortality was observed during this period.

After acclimation, mussels were subject to a gradual temperature rise of 1°C per day during 8 days (from 16°C to 24°C in fall, from 12°C to 20°C in winter from 20°C to 28°C in summer) and maintained at 24°C in fall, 20°C in winter and 28°C in summer for 3 weeks (Fig 1). Because the main goal of our study was to compare responses to gradual warming between the mussels from clean and polluted areas, we chose not to include an experimental group maintained at the constant temperature in the laboratory. An earlier study showed that laboratory maintenance for 14 days at the constant temperature does not significantly change the season-specific levels of the studied biomarkers compared to time zero samples [6]. Mussels (n = 5) were retrieved at days 0, 4, 8 (during temperature rise) and at days 14 and 27 (after acclimation to elevated temperature; 20, 28 or 24°C) except for biochemical analyses in which day 4 was not included. Studied biomarkers were determined individually for each mussel. The digestive gland was dissected out and halved. One half was placed on plastic cryovials and directly frozen into liquid nitrogen. The second half was fixed separately in 4% formaldehyde containing 0.1 M phosphate buffer at 4°C for 2 days. The posterior adductor muscle tissue was dissected out, placed into cryovials and directly frozen in liquid nitrogen. Frozen samples were stored at -80°C until required for analysis. Blind-coded labels were used in order to avoid operator’s subjectivity.

### Oxidative stress biomarkers

Total Antioxidant Capacity (TAOC) measurement is based on the suppression of the absorbance of radical cations of 2,2′-azinobis (3-ethylbenzothiazoline 6-sulfonate; ABTS) by antioxidants in the test sample when ABTS incubates with a peroxidase (metmyoglobin) and H_2_O_2_. Posterior adductor muscle tissues were homogenized in ice cold PBS with protease inhibitors. Total antioxidant capacity was measured using colorimetric microplate assay for total antioxidant power (Oxford Biomedical Research, Oxford, MI, USA) following the manufacturer's protocol. TAOC was expressed in TROLOX equivalents against a standard and normalized for the protein content of the sample.

### Lysosomal membrane stability

Digestive gland serial cryotome sections (10 μm thick) from 5 mussels were cut in a Leica CM 3000 cryostat (Leica instruments) and stained for histochemical demonstration of N-acetyl-β-hexoxaminidase activity. Labilization period (LP) of the lysosomal membrane was established according to the time of acid labilization required to produce the maximum staining of lysosomes. The mean value, determined for 4 measurements made in each individual, was obtained for each mussel digestive gland according to standard procedures [35–37].

### Lysosomal structural changes

Digestive gland cryotome sections (8 μm thick) of 5 mussels were cut in a Leica CM 3000 cryostat and stained for histochemical demonstration of β-glucuronidase activity [35,38]. Using a 100× objective lens, five measurements were made for each individual using image analysis. The mean value of the following stereological parameters was determined for each mussel digestive gland [39]: lysosomal volume density (V*v*_L_), lysosomal surface density (S*v*_L_) lysosomal surface to volume ratio (S/V_L_) and lysosomal numerical density (N*v*_L_).

### Intracellular accumulation of neutral lipids

Digestive gland cryotome sections (8 μm thick) of 5 mussels were cut in Leica CM 3000 cryostat and stained using the method of Lillie & Ashburn’s Oil Red O at 40× magnification. Five measurements were made by image analysis (Sevisan) to calculate the volume density of intracellular neutral lipids in digestive cells (V*v*_NL_) [22].

### Histopathology and tissue-level biomarkers

Digestive gland samples fixed in phosphate-buffered 4% formaldehyde were dehydrated in a graded ethanol series and embedded in paraffin. Histological sections (5 μm thick) were cut using a rotary microtome (Leitz 1512) and stained with haematoxylin and eosin (H&E). A stereological procedure was applied to quantify the volume density of basophilic cells (V*v*_BAS_; μm^3^/μm^3^); mean epithelial thickness (MET; μm), mean luminal radius (MLR; μm) were also quantified in order to calculate the ratio MLR/MET (μm/μm) as an integrative measurement of changes in alveolus morphology [40]. Connective to diverticula (CTD) ratio was also calculated [41]. Counts were made in 3 optical fields per slide in 5 slides per sample, each slide containing an individual mussel digestive gland. Slides were viewed at 40× magnification using a drawing tube attached to a Leitz Labrolux S Optiphot microscope. A Weibel graticule (multipurpose system M-168) [42] was used, and hits of basophilic and digestive cells, luminal area and connective tissue were recorded to calculate V*v*_BAS_, MLR/MET and CTD.

### Energetic biomarkers

Activities of hexokinase (HK; EC 2.7.1.1), glycogen phosphorylase (GP; EC 2.4.11), pyruvate kinase (PK; EC 2.7.1.40), phosphoenolpyruvate carboxykinase (PEPCK; EC 4.1.1.31) and cytochrome c oxidase (COX; EC 1.9.3.1) were determined, as biomarkers of changes in aerobic metabolism, mitochondrial respiration and glycolysis, using standard spectrophotometric techniques [43–45]. Posterior adductor muscle tissue was thoroughly homogenized in enzyme-specific homogenization buffer (see below) using hand-held Kontes^®^ Duall^®^ tissue grinders (Fisher Scientific, Suwanee, GA, USA). Homogenates were sonicated 3×10 s (output 7, Sonic Dismembrator Model 100, Fisher Scientific, Suwanee, GA) to ensure complete release of the enzymes, cooled on ice (1 min) between sonications and centrifuged at 16000*g* and 4°C for 25 min. The supernatant was collected and used for enzyme determination. Enzyme extracts were stored at −80°C for less than 2 weeks before activity assays. Enzyme activities were measured at 20°C using a UV—Vis spectrophotometer (VARIAN Cary 50 Bio, Cary NC, USA). The temperature of the reaction mixture was controlled within 0.1°C of the set value using a water-jacketed cuvette holder. Briefly, isolation and assay conditions for the studied enzymes are shown in S1 Table. Protein concentration was measured in the supernatant using the Bio-Rad Protein Assay kit according to the manufacturer's protocol (Bio-Rad Laboratories, Hercules, CA, USA) and specific activities were expressed as U/g protein.

### Gametogenesis and gonad histopathology

Mantle samples fixed in phosphate-buffered 4% formaldehyde were dehydrated in a graded ethanol series and embedded in paraffin. Histological sections (5 μm thick) were cut using a rotary microtome (Leitz 1512) and stained with H&E. Six gamete developmental stages were distinguished and a gonad index (GI) value was assigned to each developmental stage [46,47]: 1, resting; 1.5, early gametogenesis; 3.5, advanced gametogenesis (ripe and developing gametes at about equal proportions); 5, mature; 3.5, spawning stage (some follicles appear empty); 1.5, post-spawning stage (empty follicles). A mean GI was then calculated for each studied group. After histopathological examination at the light microscope, oocyte atresia prevalence (OAP) and intensity (gross estimate) were systematically recorded together with other anomalies (e.g., necrosis, small sized mature follicles).

### Derived parameters and statistics

SPSS 13.0 for Windows (SPSS inc, Chicago, IL, USA) was employed for statistical analysis. For biomarkers, the analysis was performed using generalized linear model analysis of variance (ANOVA) followed by *post hoc* procedures (Duncan’s *post hoc*) or in the case of PK, PEPCK, PK/PEPCK, COX, HK and GP (Fisher’s Least Significant Difference test for unequal N). Differences between sites, as well as seasonal differences at Time 0 (*T*_*0*_) were established after student’s *t*-test for each population. Prior to analysis assumptions for the normality of data distribution (Kolmogorov-Smirnov test) and homogeneity of variances (Levene's test) were tested, if any of the assumptions was violated, the data were log transformed. In the case of LP, V*v*_L_, S/V_L_, N*v*_L_ V*v*_NL_ and GI, Dunn’s *post hoc* was performed after non parametric ANOVA (Kruskal-Wallis). In addition, Mann-Whitney *U*-test was performed in order to check for significant differences between sites, as well as seasonal differences in *T*_*0*_ for each population. Differences between groups in the prevalence of gametogenic stages and oocyte atresia were studied by χ^2^ test. Linear regression models were derived for the effect of elevated temperature and time on each biomarker. Best fit models were established after *F*-test and significance of regression coefficient was determined after *t*-test. The analysis of covariance (ANCOVA) and Bonferroni’s *post-hoc* test were used to compare slopes was performed. In all cases a 95% significance level (*p*<0.05) was established.

## Results

### Natural mussels' health condition

**Fig 2:**
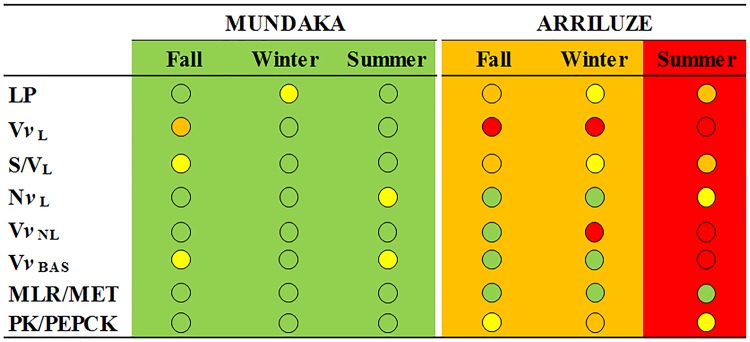
Ecosystem Health Condition Chart (EHCC) [17]. EHCC performed using eight biomarkers (LP, VvL, S/VL, NvL, VvNL, VvBAS, MLR/MET, PK/PEPCK) measured in mussels collected from Mundaka and Arriluze in fall, winter and summer. Each biomarker was assigned a grading scale according to the environmental degradation represented by a colour spot. Background colour corresponds to the environmental health condition of each season (determined by the integration of individual biomarkers). Green = "good ecosystem health condition"; yellow = "tolerable ecosystem health condition"; orange = "delicate ecosystem health condition"; red = "bad ecosystem health condition".

**Fig 3:**
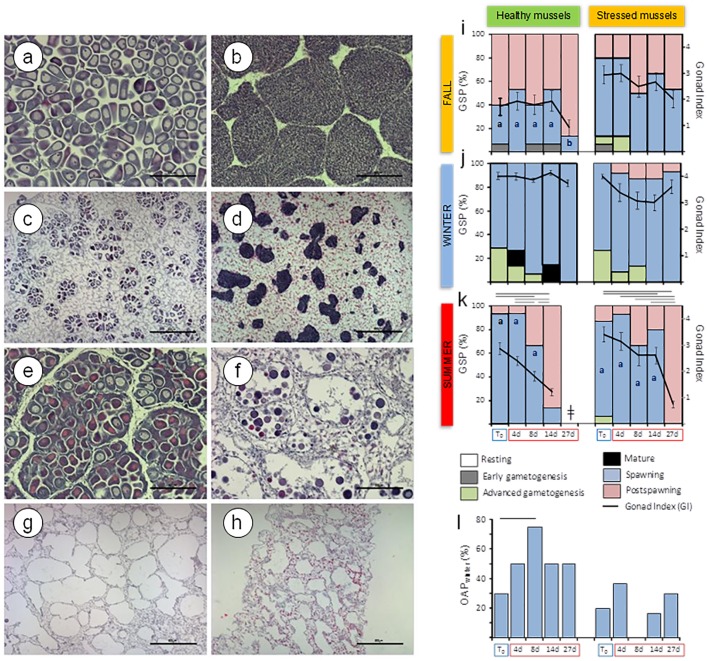
Gonad histology and gametogenesis. (a-h) Paraffin sections stained with hematoxylin-eosin of the gonad of healthy mussels from Mundaka and stressed mussels from Arriluze subjected to experimental temperature elevation: (a-b) Mature follicles in healthy female (a) and male (b) in winter. (c-d) Small sized follicles with mature gametes at post-spawning stage in stressed female (c) and at spawning stage in stressed male (d), in fall. (e) Extensive oocyte atresia in healthy female in winter. (f) Putative necrotic oocytes (strongly stained or eosinophilic hyaline cytoplasm) in healthy female subjected to experimental temperature elevation for 14 d in summer. (g-h) Empty gonad follicles after massive spawning induced by experimental temperature elevation in healthy (g) and stressed (h) mussels. Scale bar (a, b, e, f) = 200 μm; scale bar (c, d, g, h) = 50 μm. Gametogenic stages and GI in fall (i), winter (j) and summer (k), and oocyte atresia in winter (l) in healthy and stressed mussels subjected to experimental temperature elevation. Vertical segments represent standard errors. Horizontal segments above bars indicate significant differences in GI between experimental times within each mussel population, after the Kruskall Wallis test and Dunn’s *post hoc* (*p*<0.05). Asterisks indicate, within each mussel population, significant differences between T_0_ and experimental times in the prevalence of the "spawning" stage and oocyte atresia, after the χ^2^ test (*p*<0.05). GSP: gametogenic stage prevalence (%); OAP_winter_: oocyte atresia prevalence in winter (%).

Several biomarkers in the chronically stressed population (Arriluze) surpassed regional reference values for mussels from the Basque Coast (Southern Bay of Biscay [24–26,48–52] and consequently the Ecosystem Health Condition Chart (EHCC), based upon the use of mussels as sentinels of ecosystem health disturbance [17], revealed a '*delicate ecosystem health condition*' in winter and fall and a '*bad ecosystem health condition*' in summer (Fig 2). In contrast, lysosomal and tissue-level biomarkers in the healthy mussel population (Mundaka) were within established reference values all over the study period corresponding to the EHCC '*good ecosystem health condition*' (Fig 2). In addition, whilst healthy mussels presented one spawning peak, stressed mussels showed continuous spawning, as previously reported [51,53]. Thus, in fall, healthy mussels were in the post-spawning stage while stressed mussels were at spawning stage (Fig 3i; Table 1). Moreover, unlike healthy mussels that had only a few small mature follicles in fall, stressed mussels exhibited extensive "imperfect ripeness" (Fig 3c and 3d), a condition apparently associated with unremitting advanced gametogenesis [53–55].

**Fig 4:**
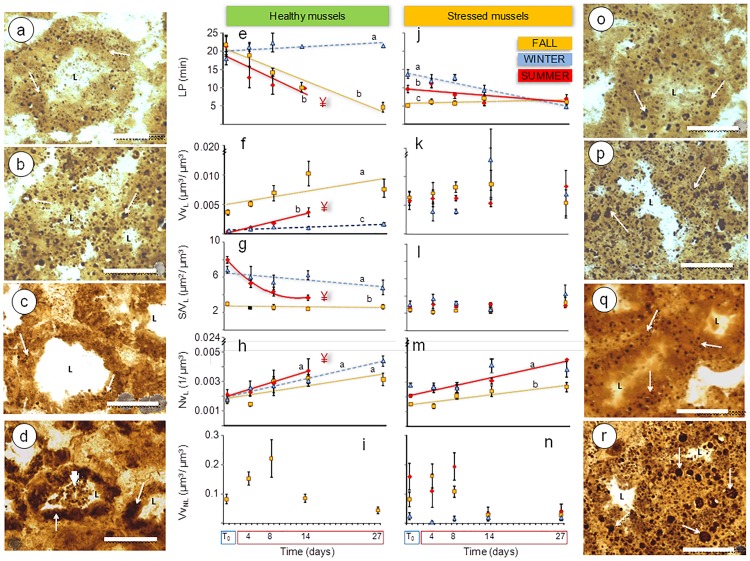
Lysosomal biomarkers. (a-d and o-r) Cryotome sections where the cytochemical localization of β-glucuronidase reveals digestive cell lysosomes in digestive gland alveoli: (a) healthy mussels from Mundaka in fall at T_0_ and (b) at day 27 (note lysosomal enlargement); (c) healthy mussels in summer at T_0_ and (d) at day 14 (note differences in lysosomal size between fall and summer and lysosomal enlargement after temperature elevation); (o) stressed mussels from Arriluze in fall at T_0_ and (p) at day 27; and (q) stressed mussels in summer at T_0_ and (r) at day 27 d (note difference in lysosomal size between localities and lysosomal enlargement after temperature elevation). LP, V*v*_L_, S/V_L_, N*v*_L_ and V*v*_NL_ in digestive cells of healthy mussels (e-i) and stressed mussels (j-n) subjected to experimental temperature elevation in fall (black square), winter (black triangle), summer (black rhombus). Vertical segments represent standard error. Significant linear regressions (S4 Table) are represented by dotted yellow line in fall, dashed blue line in winter and solid red line in summer. Significantly different regression models are indicated by different letters (*a*, *b* and *c*) after ANCOVA (*p*<0.05). ¥: 100% mortality at day 27. Scale bars = 50 μm. L: Lumen. Arrows: digestive cell lysosomes. Arrowhead: excreted lysosomes in the lumen.

Total antioxidant capacity (TAOC) varied with season in both mussel populations but higher values were recorded in healthy mussels than in stressed ones (Table 1). Likewise, lysosomal biomarkers significantly varied among seasons and between populations (Table 1). Typically, lysosomal responses to environmental stress involve increased lysosomal size, membrane destabilization and changes in lysosomal content [22]. Changes in lysosomal size are often quantified as changes in the volume density (Vv_L_), surface density (Sv_L_), surface-to-volume ratio (S/V_L_) and numerical density (Nv_L_) of the lysosomes. A shorter labilization period (LP) reveals suppressed membrane stability, while changes in the volume density of intracellular neutral lipids in digestive cells (Vv_NL_) are indicative of changes in lysosomal contents [22]. LP was always above 18 min in healthy mussels and significantly lowered in stressed ones (Table 1). V*v*_L_ was higher in the fall (reflecting large and abundant digestive cell lysosomes) than in the winter and summer in healthy but not in stressed mussels (Table 1). V*v*_L_ values were within Basque Coast baseline [24,48,50] in healthy mussels but not in stressed mussels. Whilst healthy mussels had overall low V*v*_L_ values (<0.001 μm^3^/μm^3^) which showed marked seasonality with abundant small lysosomes in winter and summer and large lysosomes in the fall, stressed mussels showed high V*v*_L_ (indicative of large lysosomes) throughout the year (Fig 4, Table 1). Intracellular neutral lipids were always abundant in stressed mussels (with especially high V*v*_NL_ in summer) unlike in healthy mussels in which considerable levels of V*v*_NL_ were only recorded in fall.

**Fig 5:**
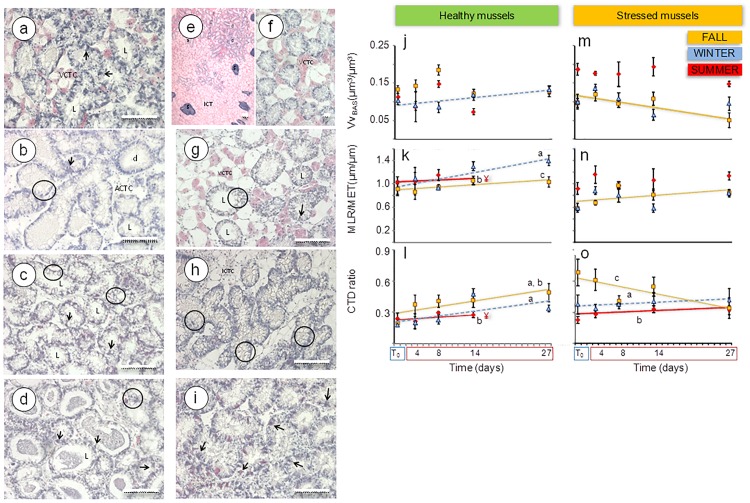
Tissue level-biomarkers. (a-i) Paraffin sections stained with haematoxylin-eosin of the digestive gland of healthy mussels from Mundaka and stressed mussels from Arriluze subjected to experimental temperature elevation: (a) healthy mussels in fall at T_0_ and (b) in winter at T_0_ (note extensive vacuolization); (c, d) healthy mussels in summer at day 14 (note extensive vacuolization in 2c and epithelial thinning in 2d); (e,f) stressed mussels in fall at T_0_ (note copious ICT in 2e) and (g) at day 27 (note extensive vacuolization and epithelial thinning); (h) stressed mussels in winter at T_0_ (note extensive vacuolization) and (i) in summer at day 27 (note high occurrence of basophilic cells). V*v*_BAS_, MLR/MET and CTD ratio of healthy mussels (j-l) and stressed mussels (m-o) subjected to experimental temperature elevation in fall (black square), winter (black triangle), summer(black rhombus). Vertical segments represent standard error. Significant linear regressions (S4 Table) are represented by dotted yellow line in fall, dashed blue line in winter and solid red line in summer. Significantly different regression models are indicated by different letters (*a*, *b* and *c*) after ANCOVA (*p*<0.05). ¥: 100% mortality at day 27. Scale bars = 100 μm. a: alveoli; d: duct; g: gonad; ICT: Interstitial connective tissue; VCTC: Vesicular connective tissue cells; ACTC: Adipogranular connective tissue cells; L: lumen; arrows: basophilic cells; circles: vacuolization.

In healthy mussels, digestive gland histology was normal in the fall and summer (Fig 5a) but massive vacuolization with obliterated lumens was observed in winter (Fig 5b). In contrast, swelling of digestive cell apex in the fall (Fig 5f) and massive vacuolization in winter (Fig 5h) were found in stressed mussels. The volume density of basophilic cells (Vv_BAS_) reflects changes in the cell type composition in the digestive gland epithelium and the ratio MLR/MET (mean epithelial thickness divided by mean luminal radius) is an integrative measure of changes in morphology of digestive alveoli [26,51]. The connective-to-diverticula (CTD) ratio reflects the structural integrity of the digestive gland tissue [41]. In stressed mussels, basophilic cells were particularly abundant in summer (Fig 5i) and the interstitial connective tissue content was highest in the fall (Fig 5e). Overall, V*v*_BAS_, MET, MLR, MLR/MET and CTD ratio were within the baseline levels and did not vary among seasons in healthy mussels, whereas seasonal variability was apparent in stressed mussels that showed higher MET, lower MLR/MET and CTD ratio than healthy mussels (Table 1).

In contrast, no seasonal variation was observed in pyruvate kinase (PK), phosphoenol pyruvate carboxykinase (PEPCK) and cytochrome c oxidase (COX) activities in stressed mussels (Table 1). In healthy mussels, PK and PEPCK activities were elevated in the winter and COX activity was elevated in the summer (Table 1). PK/PEPCK ratio was higher in healthy mussels compared to their stressed counterparts and did not vary seasonally. Hexokinase (HK) activity was maximal in the winter and minimal is the summer and fall in both studied populations, while glycogen phosphorylase (GP) activity was maximal in the fall in the healthy population and in the summer in the stressed one (Table 1). The responses elicited by the same experimental temperature elevation (+8°C above the ambient) in the fall, winter and summer were different depending on the initial condition of the mussels (stressed *vs*. healthy) and the acclimation temperature (20, 24 or 28°C) of each season (S2 Table).

### Healthy mussels' response to warming

**Fig 6:**
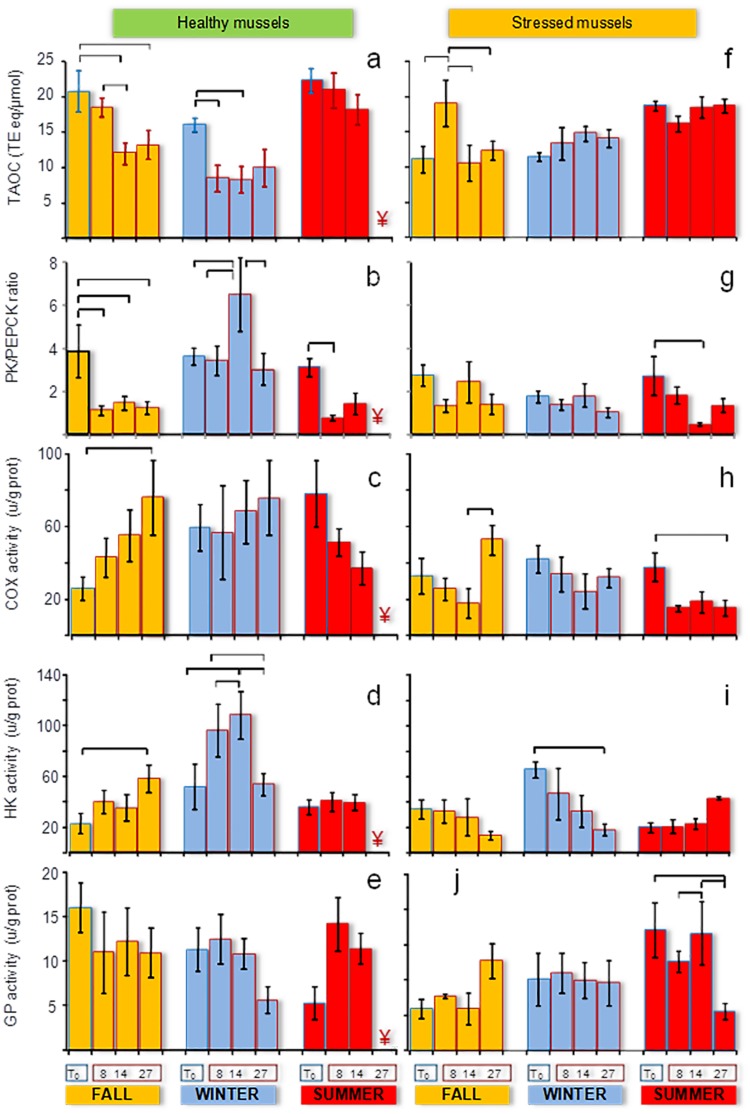
Oxidative stress and energetic biomarkers. TAOC, PK/PEPCK ratio and COX, HP and GP enzyme activities of healthy mussels from Mundaka (a-e) and stressed mussels from Arriluze (f-j) subjected to experimental temperature elevation in fall (yellow), winter (blue) and summer (red). Vertical segments represent standard error. Horizontal segments indicate significant differences within each group after one way ANOVA and LSD *post hoc* (*p*<0.05). ¥: 100% mortality at day 27.

In the fall, seawater warming from 16°C to 24°C enhanced both aerobic and anaerobic pathways as well as mitochondrial respiration, but the aerobic scope was largely reduced (PK/PEPCK ratio dropped to very low levels). A significant transient increase was induced in PEPCK activity and consequently the PK/PEPCK ratio dropped to values close to 1 at day 27 (Fig 6b). At the same time, COX and HK activity increased and GP activity was slightly suppressed (Fig 6c–6e). TAOC was inhibited in healthy mussels in the fall (Fig 6a; S3 Table). LP decreased linearly with the warm exposure reaching extremely low values (<5 min) after 27 days at 24°C (Fig 4e; ANCOVA, *p*<0.05; S4 Table). The highest V*v*_L_ values were recorded in the fall after 27 days at the elevated temperature (ANCOVA, *p*<0.05) but S/V_L_ values were not affected by warming (Fig 4f and 4g). In V*v*_NL_, a biphasic response was observed in the fall, with a remarkable initial increase until day 8 followed by a gradual decrease, particularly marked at day 27 (Fig 4i). Lysosomal responses were accompanied by thinning of the digestive gland epithelium and retraction of the digestive alveoli, shown by a transient increase in V*v*_BAS_, linear increase in MLR/MET and connective-to-diverticula (CTD) ratio and a linear decrease in MET (y_(fall)_ = 12.93–0.08x; *F*_(1,19)_ = 7.026, *p* = 0.016; Fig 5j–5l). Spawning was boosted so that most mussels were at the post-spawning stage by day 27 (Fig 3i).

In winter, gradual warming from 12°C to 20°C intensified aerobic metabolism, mitochondrial respiration and glycolysis, as indicated by a transient increase in PK/PEPCK ratio (Fig 6b) and elevated COX and HK activities (Fig 6c and 6d). TAOC dropped as the temperature rose from 12 to 20°C after day 8 and remained low after 3 weeks at 20°C (Fig 6a; S3 Table). LP remained above 20 min throughout the exposure (Fig 4e); however, digestive cell lysosomes were slightly more abundant (Fig 4h) and glycogen breakdown was active for 14 days after warming (Fig 6e). Modest changes in the size and number of digestive cell lysosomes (Fig 4g and 4h) indicate changes in heterophagic processes. Temperature elevation induced a linear increase in V*v*_BAS_ (Fig 5j; S4 Table). MLR/MET and CTD ratio increased linearly during warm exposure (Fig 5k and 5l; S4 Table), concomitant with a decrease in MET (y_(winter)_ = 13.38–0.13x; *F*_(1,19)_ = 15.996, *p* = 0.001). Despite the overall significant trend, V*v*_BAS_ and MLR/MET remained close to the baseline levels (Table 1) except for a notable rise in MLR/MET at day 27. Extensive oocyte atresia (>50%) was induced by prolonged warming (Fig 3e, 3k and 3l).

In summer, temperature elevation from 20°C to 28°C suppressed COX activity and enhanced PEPCK and GP activities (Fig 6b, 6c and 6e). The ratio of PK/PEPCK dropped to values close to 1, indicating a decrease of the aerobic scope that persisted after 8–14 days of warming (no data for 27 days could be obtained due to 100% mortality). TAOC levels remained stably high, similar to the values found at T_0_ (Fig 6a). Lysosomal enlargement and membrane destabilization were impressive (Fig 4d and 4e–4h), accompanied by the digestive cell vacuolization and atrophy of the digestive alveoli (Fig 5c and 5d). LP and S/V_L_ decreased and V*v*_L_ and N*v*_L_ increased in response to prolonged warming (Fig 4e–4h; ANCOVA, *p*<0.05; S4 Table). V*v*_L_ values were 10-fold higher after gradual warming than at T_0_, compared to only 2-fold enlargement in the winter. Massive spawning occurred by day 14 (Fig 3k), along with the gonad resorption and gamete necrosis (Fig 3f).

### Stressed mussels' response to warming

In the fall, gradual temperature increase from 16°C to 24°C enhanced both aerobic (COX activity) and anaerobic (PEPCK activity) metabolism (Fig 6g and 6h). GP and HK activities remained at the baseline levels throughout 27 days of warming (Fig 6i and 6j). A biphasic response was induced in V*v*_NL_, revealing transient mobilization of neutral lipids (Fig 4n). There was a notable (albeit non-significant trend) of the warming-induced decrease in the aerobic scope with PK/PEPCK ratio reaching the value of <1 after 27 days of exposure (Fig 6g; S3 Table). Lysosomal enlargement and membrane destabilization were already profound at T_0_ (LP = 5 min; Fig 4j) but prolonged warming caused no additional effect in LP. Furthermore, a transient increase in V*v*_L_ at day 14 and a linear increase in N*v*_L_ were recorded after gradual warming (Fig 4k and 4m, S4 Table). Overall, the range of variation in lysosomal biomarkers at day 27 was narrower than in healthy mussels (Fig 4a–4d *vs*. Fig 4o–4r). As a result, differences in responses between the two studied mussel populations vanished at day 27. In stressed mussels collected in the fall, temperature elevation enhanced digestive cell vacuolization and swelling, as well as atrophy of the digestive alveoli, so that the lumen was occupied by swollen apical portions and vacuoles of digestive cells (Fig 5g). In parallel, basophilic cells were less conspicuous and V*v*_BAS_ was reduced (Fig 5m). However, the most remarkable observation was the linear reduction in CTD ratio with temperature elevation, revealing that the interstitial connective tissue was gradually shrinking (Fig 5o; S4 Table). The gametogenic cycle appeared to be arrested, as early and advanced gametogenesis stages vanished after 8 days and progression towards post-spawning stages was limited (Fig 3i).

In winter, stressed mussels showed increased PEPCK activity and slightly reduced aerobic scope in response to the long-term warming (Fig 6g). COX activity, indicative of mitochondrial respiration, remained unchanged (Fig 6h) but HK activity was strongly inhibited (Fig 6i). Active glycogen breakdown (GP activity) was high in stressed mussels at T_0_ and remained so after temperature elevation (Fig 6j). TAOC did not change (Fig 6f). A significant linear decrease in LP was recorded over experimental time (ANCOVA; *p*<0.05; Fig 4j; S4 Table), such that LP reached 10 min by day 14 and 5 min by day 27 (Fig 4j). However, other lysosomal responses (Fig 4k–4n) were less variable: V*v*_BAS_ and MLR/MET showed no clear trend (Fig 5m and 5n), but a slight linear increase in CTD was observed (Fig 5o; S4 Table). Temperature elevation did not affect neutral lipid accumulation in the digestive cells (Fig 4n), which was low in this season (Table 1). Spawning was moderately induced, as shown by certain increase in the prevalence of post-spawning stages at the expense of advanced gametogenesis progression (Fig 3j).

In summer, experimental temperature elevation from 20°C to 28°C enhanced anaerobic metabolism and transiently lowered the aerobic scope (indicated by a decrease in PK/PEPCK ratio after 8–14 days of exposure) as well as suppressed mitochondrial capacity (shown by decreased COX activity) in stressed mussels (Fig 6f; S3 Table). However, at a later stage (27 days of exposure) the anaerobic metabolism (indicated by PEPCK activity) was suppressed in parallel with partial recovery of aerobic scope. Prolonged warming also suppressed GP activity but did not affect HK activity (Fig 6g–6i). V*v*_NL_ was decreased due to the prolonged (27 days) warming (Fig 4n; S4 Table). TAOC was high in stressed mussels at T_0_ and remained unchanged after temperature elevation (Fig 6f). In contrast, prolonged (14 days) temperature elevation (28°C) led to considerable lysosomal enlargement and membrane destabilization as well as mobilization of neutral lipids beyond the high initial values of these parameters in stressed mussels. A significant linear decrease in LP was recorded over experimental time (Fig 4j; ANCOVA; *p*<0.05) together with a linear increase in N*v*_L_ (Fig 4m, S4 Table). High V*v*_BAS_ was recorded at all sampling times (Fig 5m) and some basophilic cells were apparently necrotic beyond day 14 (Fig 5i). Massive spawning occurred by day 27 (Fig 3k).

## Discussion

### Natural mussels' health condition

Ecosystem Health Condition Chart (EHCC) [17] revealed a '*delicate ecosystem health condition*' in winter and fall and a '*bad ecosystem health condition*' in summer in the chronically stressed population (Arriluze), and a '*good ecosystem health condition*' in the healthy mussel population (Mundaka). Unlike healthy mussels, stressed mussels showed attenuated seasonality in reproduction as evidenced by continuous spawning [51,55]. Accordingly, with the exception of the HK activity that showed similar seasonal dynamics in the two studied populations (peaking in winter), the seasonality observed in other biomarkers in the healthy mussel population was largely altered in the stressed mussel population. The healthy mussel population had comparable aerobic scope and digestive gland condition throughout the seasons but showed marked seasonality in lysosomal biomarkers, reserve storage and antioxidant capacity, as previously reported [25,27,50–52,56]. V*v*_L_ values were higher in the fall than in winter and summer, high PK and PEPCK activity values were recorded in winter and high COX activity values in summer unlike in stressed mussels in which such seasonality was not observed. In contrast, V*v*_BAS_, MET, MLR, MLR/MET, CTD ratio and PK/PEPCK did not vary amongst seasons in healthy mussels but showed notable seasonality in stressed mussels. Besides, TAOC was always higher in healthy mussels than in stressed ones, LP was always above 18 min in healthy mussels and significantly lowered in stressed ones; and V*v*_NL_ was especially high in summer in stressed mussels. GP activity was maximal in the fall in the healthy population but in summer in the stressed one.

Overall, the stressed mussel population exhibited disturbed health status together with altered seasonality in biomarkers and gametogenesis, which could be a consequence of chronic environmental stress combined with continuous nutrient supply as the result of urban and harbour pollution [25,28,29,48,51,55]. Indeed, the seasonal pattern of biomarkers and the gamete development cycle, which are driven by locally relevant factors such as temperature and food availability, may differ between neighbouring populations, the seasonal range of variation being narrower in stressed populations than in healthy ones [25,27,48,51,52, 56–60]. Besides, the responses elicited by the identical experimental temperature elevation (+8°C above the ambient) in fall, winter and summer were different depending on the initial condition of the mussels (stressed *vs*. healthy) and the acclimation temperature (20, 24 or 28°C) of each season.

### Healthy mussels' response to warming

In fall, seawater warming enhanced both aerobic and anaerobic pathways as well as mitochondrial respiration, but the aerobic scope was largely reduced (PK/PEPCK ratio dropped to very low levels). Mussels are known to respond to temperature elevation by altering metabolic rates and enzyme activities [61]. Thus, it is likely that the metabolic balance was impaired due to their limited physiological capacity to match a high oxygen demand at elevated temperatures (24°C) leading to a stronger reliance on anaerobic pathways [62]. Up-regulation of the metabolic enzyme activities appeared insufficient to cope with the energy demand imposed at 24°C and mussels entered a thermally stressed condition [63]. The response profile showed typical signs of stress response [17,21], such as moderate lysosomal enlargement and thinning of the digestive gland epithelia together with remarkable lysosomal membrane destabilization. These stress signals were accompanied by transient mobilization of neutral lipids and enhanced glycolysis, indicative of reserve mobilization and augmented metabolic activity [5,8]. Yet, the antioxidant capacity was inhibited rather than stimulated, which could be the consequence of augmented mitochondrial respiration and/or the result of peroxidative stress, as previously reported in stressed mussels [19,56,64]. Moreover, autophagy was apparently promoted, which may have counteracted oxidative processes such as lipid peroxidation [21]. Lysosomal enlargement and membrane destabilization were remarkable and were accompanied by thinning of digestive gland epithelium and retraction of digestive alveoli as a result of enhanced autophagy, as reported in mussels subjected to environmental stress, e.g. pollution [6,21,39,65–67]. Ultimately, mussels' health status deteriorated after prolonged exposure to 24°C (indicated by extremely low LP at day 27), and spawning was induced by day 27. Although no mortality was observed, the elicited biological responses would ultimately impinge on growth and reproduction resulting in time-limited survival, as demonstrated previously [5,6].

In winter, gradual warming intensified aerobic metabolism, mitochondrial respiration and glycolysis, as indicated by a transient increase in PK/PEPCK ratio and in COX and HK activities. This indicates that metabolic rate initially increased at increasing temperatures to compensate for elevated energy demand [68–70] but aerobic metabolism, mitochondrial respiration and glycolysis returned to initial levels by day 27. This response profile could reflect metabolic adaptation to 20°C and/or the positive effects of the improved feeding conditions in the laboratory in comparison with the winter field conditions. Overall, gradual warming did not lead to oxidative stress and lysosomal membrane destabilization in winter. TAOC dropped as the temperature rose from 12 to 20°C after day 8 and remained low after 27 days at 20°C. This suggests that the antioxidant capacity reached a new steady-state, as reported in clams and oysters after long-term moderate warming (+5°C) [54]. LP remained above 20 min, indicating that mussels were in good condition [23]. However, digestive cell lysosomes were slightly more abundant and glycogen breakdown was active. Modest changes in the size and number of digestive cell lysosomes, known to be minuscule and dormant in winter [25,50,51], would indicate an intensification of intracellular digestion [23,50,71] in response to augmented metabolic activity at higher temperature and in the presence of additional food supply. In consequence, active glycogen breakdown was replaced by other energy sources (e.g., food assimilation) after acclimation to 20°C. In agreement, temperature elevation induced a moderate thinning of the digestive gland epithelium that would result from intensification of intracellular digestion, with less digestive diverticula in "holding" phase during the normal cycle of the phasic digestion [72]. In addition, extensive oocyte atresia, a symptom of reproductive impairment reported to occur upon environmental insult [53,55,73], was recorded. Gonad resorption would provide a surplus energy source to cope with extra metabolic demand resulting from temperature elevation [59,60].

In summer, temperature elevation inhibited COX activity and enhanced PEPCK and GP activities, indicating a switch to anaerobic metabolism and active glycogen breakdown [5,8,18]. However, energetic requirements were not fulfilled as the PK/PEPCK ratio dropped to values close to 1, reducing aerobic scope to a minimum that persisted beyond day 8 of exposure to 28°C. Such extremely reduced aerobic scope suggests that the critical temperature was surpassed and survival was time limited [63], supported by anaerobic metabolism and protective mechanisms such as heat shock protein induction and antioxidant defence enhancement [5,16,69,70]. As TAOC levels remained stably high, it is conceivable that antioxidant capacity was near its upper limit in this season and thus temperature elevation was ineffective in further enhancing antioxidant enzyme activities beyond their initial levels. Remarkable lysosomal enlargement and membrane destabilization were accompanied by digestive cell vacuolization and digestive alveolus atrophy. This response profile reflects disturbance of feeding and digestion, as reported in other *Mytilus* populations [5,69,74], and severe health impairment [21,23,24,26] at seawater temperatures in the 25–28°C range. Likewise, early massive spawning (day 14), gonad resorption and gamete necrosis were signals of severe biological harm [53,55]. Thus massive mortality (100%) was finally recorded by day 27 at 28°C, which also occurred in Mediterranean *M*. *galloprovincialis* at 26°C [5].

The present results confirm previous findings obtained in healthy mussel populations [6]. Overall, responses of mussels to temperature elevation (8°C above the ambient temperature) depend on the endpoint temperature rather than on the season; indeed, for some biomarkers (e.g. LP) similar values were recorded at 24°C in fall and summer. Thus, at temperatures up to 20°C no sign of thermal stress is evident in any season. Even in winter, acclimatization rather than stress is elicited after 2–3 weeks at 20°C (Table 2); nevertheless, this acclimatization comes at a cost of diminished reproductive capacity in healthy mussels. At 24°C, both in fall and summer, healthy mussels experience thermal stress in the short-term but may enter a time-limited survival period after long-term warming (Table 2). At 28°C in summer, healthy mussels are able to survive in the short-term but they are close to their upper temperature limit and massive mortality unavoidably occurs during the long-term exposures (Table 2).

**Table 2:** Summary of biological responses elicited by prolonged temperature elevation at different season in healthy mussels and chronically stressed mussels. Main different responses between healthy and stressed mussels are highlighted in **“bold”** *plus* **#.**

SEASON	ENDPOINT	Healthy mussel population	Stressed mussel population
**FALL**	**Aerobic scope #**	Reduced	Unchanged
	Mitochondrial respiration	Enhanced	Enhanced
	**Glycolysis #**	Enhanced	Unchanged
	**Glycogen breakdown #**	Enhanced	Enhanced (late)
	**Antioxidant capacity #**	Reduced	Transiently enhanced
	Lysosomal responses	Enhanced (autophagy)	Beyond T_0_ (autophagy)
	Accumulation/mobilization of neutral lipids	Enhanced	Enhanced
	**Epithelial thinning & cell type composition #**	Slight thinning	Moderate cell swelling
	**Connective-to-Digestive ratio #**	Increased	Decreased
	**Reproduction (gametogenesis) #**	Spawning induction (late)	Gametogenic cycle arrest
	Mortality	Unchanged	Unchanged
	**RESPONSE PROFILE TYPE**	**Thermal stress: time-limited survival**	**Thermal stress: delicate health condition**
**WINTER**	**Aerobic scope #**	Transiently increased	Unchanged
	Mitochondrial respiration	Unchanged	Unchanged
	**Glycolysis #**	Enhanced (transient)	Unchanged
	**Glycogen breakdown #**	Enhanced (late)	Supressed
	**Antioxidant capacity #**	Reduced	Constantly moderate
	**Lysosomal responses #**	Enhanced (heterophagy)	Enhanced (stress)
	Accumulation/mobilization of neutral lipids	Unchanged	Unchanged
	**Epithelial thinning & cell type composition #**	Phasic digestion	Unchanged
	Connective-to-Digestive ratio	Unchanged	Unchanged
	**Reproduction (gametogenesis) #**	Oocyte atresia (resorption)	Spawning induction (moderate)
	Mortality	Unchanged	Unchanged
	**RESPONSE PROFILE TYPE**	**Acclimation to 20°C & food**	**Thermal stress: low-to-moderate**
**SUMMER**	**Aerobic scope #**	Strongly suppressed	Transiently suppressed
	Mitochondrial respiration	Reduced	Reduced
	Glycolysis	Enhanced	Enhanced
	**Glycogen breakdown #**	Enhanced	Enhanced (transient)
	Antioxidant capacity	Constantly high	Constantly high
	Lysosomal responses	Enhanced (autophagy)	Enhanced (autophagy)
	Accumulation/mobilization of neutral lipids	Enhanced (transient)	Enhanced (transient)
	**Epithelial thinning & cell type composition #**	Extreme cell swelling	High cell swelling
	Connective-to-Digestive ratio	Unchanged	Unchanged
	**Reproduction (gametogenesis) #**	Spawning induction (early) and necrosis(resorption)	Spawning induction (late)
	**Mortality #**	100%	20%
	**RESPONSE PROFILE TYPE**	**Thermal stress: sub-acute lethality**	**Thermal stress: metabolic depression**

### Stressed mussels' response to warming

In our present study, stressed mussels were maintained in seawater collected from Arriluze and therefore remained exposed to the same pollutants as in their source locality. Thus, thermal stress interacted with other stress sources that modified the amplitude of the thermal stress response. For instance, gradual warming did not cause any consistent change in TAOC levels, which were always high in all seasons, indicating that stressed mussels were adapted to oxidative stress pressure [27,56].

In the fall, seawater warming gradually enhanced both aerobic (COX activity) and anaerobic (PEPCK activity) metabolism. Initial enhancement of mitochondrial respiration was likely accompanied by an increase in ventilation rate that would boost pollutant uptake, thus stimulating toxicity and imposing additional energy expenses for detoxification processes [8]. Accordingly, glycogen breakdown was slightly activated together with a transient mobilization of neutral lipids, and aerobic scope was maintained at the stable albeit relatively low levels. Lysosomal enlargement and membrane destabilization were already marked at T_0_ and did not respond to prolonged warming with the exception of transient changes in V*v*_L_ and a linear increase in N*v*_L_ along the experimental time. Conversely, tissue-level biomarkers and histopathology of the digestive gland confirmed the onset of general stress [26]. Moreover, lipid mobilization, glycogen breakdown and connective tissue depletion (severe reduction in CTD ratio [41]) revealed that additional energy resources were required to counteract sustained temperature elevation to 24°C [16,61]. In agreement, the gametogenic cycle was seemingly arrested, suggesting that the physiological trade-off occured to divert energy from growth and reproduction towards maintenance [59]. Consequently, stressed mussels reached a “delicate condition” state by day 27 (time limited survival [5,6]).

In winter, seawater gradual warming led to enhanced PEPCK activity, inhibition of HK activity and moderately reduced aerobic scope but COX and GP activities remained unchanged. Overall, stressed mussels appeared to face thermal stress at 20°C in winter, with survival based on anaerobic metabolism and activation of cellular protection mechanisms [5,8,18,61,63]. Although TAOC did not change, lysosomal membrane destabilization was elicited, indicating additional cellular stress [21,23]. Notably, such a general stress signal was not evident in other lysosomal responses or in tissue-level biomarkers [22–26,48]. This irresponsiveness was most likely due to the extensive vacuolization and swelling of the digestive cells that was recorded at T_0_ and remained unchanged after temperature elevation.

In summer, stressed mussels presented relatively high aerobic scope, low HK activity and high GP activity at T_0_, indicating that glycogen was the main energy source during this season [8,12]. Seawater gradual warming provoked a biphasic response: early enhancement of anaerobic metabolism and lipid mobilisation was accompanied by a reduction in mitochondrial respiration and aerobic scope and further on followed by suppression of anaerobic metabolism and partial recovery of aerobic scope and GP activity. Thus, it seems that prolonged seawater warming in summer resulted in metabolic depression in stressed mussels, which is a protection mechanism in *Mytilus* spp. to prevent excessive energy demand at elevated temperatures [75]. Stressed mussels relied on lipid and glycogen stores to cope with energy requirements and to maintain protective mechanisms at their maximum capacity at 28°C at least until day 14 when glycogen reserves were presumably depleted, as suggested by the negligible glycogen breakdown beyond day 14. Initial high TAOC values remained unchanged after temperature elevation. In contrast, lysosomal enlargement and membrane destabilization were remarkable. Negative effects of thermal stress on the lysosomal membrane stability are well documented [6,9,18,21,23,76]. Lysosomes are implicated in cell autophagy, which protects the cell by removing oxidatively damaged organelles and proteins [21]. Changes in the lysosomal morphology and number in stressed summer mussels indicate enhanced autophagy at 24–28°C. However, the protection mechanisms were overloaded eventually leading to compromised survival under the thermal stress, as mortality reached 20% at day 27. At this stage, basophilic cell necrosis as well as vacuolization and swelling of digestive cells were indicative of cell death as a result of autophagic processes [26,65–68]. Likewise, severe vacuolization and swelling were recorded in digestive cells and MLR/MET remained high after temperature elevation, though always within the baseline range [30]. Furthermore, since filtration rate and food ingestion are impaired in mussels acclimated to 28°C [70], this would increase energetic deficiency and exacerbate thermal stress.

In stressed mussels, prolonged temperature elevation to 20°C in winter caused reduction in aerobic scope, activation of anaerobic pathways and partial deterioration of health status (low-to-moderate thermal stress; Table 2), but mussels were able to survive at the expense of energy reserves. Likewise, at 24°C in fall and summer, the aerobic scope was reduced and health status severely deteriorated (Table 2), but mussels were able to survive using stored fuels (e.g. glycogen). Temperature elevation to 28°C in summer caused reduction in aerobic scope at the short-term (14 days), but metabolic depression, severe deterioration of health status, altered gametogenesis and enhanced mortality occurred in the long-term (27 days).

## Conclusions

Whereas elevated temperatures induced alterations beyond those caused by seasonal changes [27,50–52,56,58] in healthy mussels, adaptation to a polluted environment attenuated the effects of thermal stress in stressed mussels (Table 2). In comparison with healthy ones, stressed mussel populations (a) show adaptive modifications to survive in a stressed environment that may account for muted responses to thermal stress, (b) possess a high energy storage that partially contributes to alleviate thermal stress, and (c) are able to survive longer under the extremely elevated temperatures of 28°C than healthy mussel populations. As a whole, stressed mussels present lower thermal stress threshold but are considerably more tolerant to elevated temperature in the long-term than healthy mussels. Marine bivalves inhabiting polluted sites are believed to be more susceptible to thermal stress [11], which explains the lower thermal stress threshold presently found in stressed mussels in comparison with healthy mussels. However, environmental traits associated with polluted environments such as adaptation to chronic stress and eutrophication (resulting in high food availability) may alleviate these effects by providing additional energy supply to counteract the energetic requirements to deal with warming and enabling survival during prolonged thermal stress. Similar enhancement of temperature tolerance due to elevated food availability was shown in other populations from eutrophicated polluted estuaries, although this response depended on the food quality [77,78]. Moreover, gradual warming induces gonad resorption in healthy mussels, hampers the commencement of a new gametogenic cycle in fall and induces massive spawning in summer both in healthy and stressed mussels but much less markedly and at longer times in the latter. Therefore, the present study provides experimental evidence that tolerance to seasonal gradual warming may be enhanced in mussel populations chronically subject to environmental stress. This indicating that the current assumption that the interaction of multiple stressors would enhance the susceptibility of marine biota to seawater warming may not be universally true and can be modified by specific local conditions (especially energy and food availability).

## Supporting information

S1 Table Isolation and assay conditions for the biochemical determination of HK, GP, PK, PEPCK and COX enzyme activities [43–45]. (PDF)

S2 Table Summary of three-way ANOVA performed to analyze the effect of elevated temperature elevation, season, health condition and their interactions in oxidative stress and energetic biomarkers in healthy mussels (from Mundaka) and stressed mussels (from Arriluze) in fall, winter and summer. (PDF)

S3 Table Summary of Kruskall-Wallis and one-way ANOVA performed to analyze the effect of temperature elevation in studied biomarkers in healthy mussels (from Mundaka) and stressed mussels (from Arriluze) in fall, winter and summer. (PDF)

S4 Table Significant linear regressions of cell and tissue level biomarkers against experimental time after temperature elevation in fall, winter and summer for the cases of healthy mussels from Mundaka and stressed mussels from Arriluze. (PDF)
