# Supplementary material for: Chronic environmental stress enhances tolerance to seasonal gradual warming in marine mussels
Source: PLoS One. 2017 Mar 23;12(3):e0174359. doi: 10.1371/journal.pone.0174359 (PMC5363927; doi:10.1371/journal.pone.0174359)
Supplement: S1 Table — (PDF) [file pone.0174359.s001.pdf]

S1 Table. Isolation and assay conditions for the biochemical determination of HK, GP, PK, PEPCK and COX enzyme activities [43-45].

	**Homogenization buffer**	**Assay**
**HK**	10 mM pH 7.2 Tris–HCl buffer (pH 7.2), 5mM EDTA,<br>1 mM dithiothreitol (DTT),<br>0.1 mM phenylmethylsulfonyl fluoride (PMSF).	0.037 M Tris-HCl buffer (pH 7.2), 0.22 M glucose, 6.5 mM $MgCl_2$,<br>2.7 mM ATP, 0.83 mM NADH.<br>Wavelength 340 nm
**GP**	10mM Tris–HCl buffer (pH 7.2), 5 mM EDTA,<br>0.1 mM PMSF, 2 µg/mL aprotinin	50 mM potassium phosphate buffer (pH 7.2), 0.2% (w/v) glycogen,<br>1.3 mM $MgCl_2$, 0.10 mM EDTA, 0.43 mM NADP, 0.0003% (w/v) G 1,6-DiP,<br>1U G-6-PDH, 1U PGLUM, 1.6 mM AMP.<br>Wavelength 340 nm
**PK**	10 mM Tris–HCl buffer (pH 7.2), 5 mM EDTA,<br>1 mM DTT, 0.1 mM PMSF	50 mM Tris-HCl (pH7.2), 50 mM KCl, 5 mM $MgSO_4$, 1 mM ADP,<br>0.2 mg/ml NADH, 5.5U LDH, 0.5 mM PEP. Wavelength 340 nm
**PEPCK**	10mM Tris–HCl buffer (pH 7.2), 5 mM EDTA,<br>1 mM DTT, 0.1 mM PMSF.	100 mM HEPES (pH 7.2), 2.3 mM MnCl2, 0.5 mM IDP,<br>5mg/ml $KHCO_3$, 0.2 g/ml NADH, 10U MDH, 15 mM PEP.<br>Wavelength 340 nm
**COX**	10mM Tris–HCl buffer (pH 7.2), 5 mM EDTA,<br>0.1 mM PMSF, 2 µg/mL aprotinin	20 mM potassium phosphate (pH 7.0),<br>16 µM reduced cytochrome c (II), 0.45 mM *n*-dodecyl-β-d-maltoside,<br>2 µg/mL antimycin A.<br>Wavelength 550 nm
