# Supplementary material for: Chronic environmental stress enhances tolerance to seasonal gradual warming in marine mussels
Source: PLoS One. 2017 Mar 23;12(3):e0174359. doi: 10.1371/journal.pone.0174359 (PMC5363927; doi:10.1371/journal.pone.0174359)
Supplement: S2 Table — (PDF) [file pone.0174359.s002.pdf]

S2 Table. Summary of three-way ANOVA performed to analyze the effect of elevated temperature elevation (TE), season (S), health condition (HC) and their interactions in oxidative stress and energetic biomarkers in healthy mussels (from Mundaka) and stressed mussels (from Arriluze) in fall, winter and summer. *F*: Fisher's *F*; degrees of freedom are given between brackets; *p*: significance. Significant effects are indicated by bold characters (p<0.05).

Source	PK		PEPCK		PK/PEPCK ratio		COX		TAOC		HK		GP	
**TE**	$F_{(3,108)}$= 1.39	p = 0.251	$F_{(3,109)}$= 4.78	p = **0.004**	$F_{(3,105)}$= 6.41	***p* = 0.001**	$F_{(3,107)}$= 0.67	p = 0.570	$F_{(3, 76)}$= 3.65	p = **0.017**	$F_{(2,106)}$= 0.62	p = 0.606	$F_{(3, 104)}$= 1.77	p = 0.158
**S**	$F_{(2,108)}$= 24.44	***p*< 0.001**	$F_{(2,109)}$= 0.92	p = 0.402	$F_{(2,105)}$= 7.48	***p* = 0.001**	$F_{(2,107)}$= 3.49	***p* = 0.035**	$F_{(1, 76)}$= 2.47	p = 0.121	$F_{(2,106)}$= 10.93	***p*< 0.001**	$F_{(2, 104)}$= 0.51	p = 0.602
**HC**	$F_{(1,108)}$= 43.84	***p*< 0.001**	$F_{(1,109)}$= 0.01	p = 0.929	$F_{(1,105)}$= 10.35	***p* = 0.002**	$F_{(1,107)}$= 16.26	***p* = 0.001**	$F_{(1, 76)}$= 5.09	p = **0.028**	$F_{(1,106)}$= 19.88	***p*< 0.001**	$F_{(1, 104)}$= 0.08	p = 0.773
**TE x S**	$F_{(6,108)}$= 0.86	p = 0.525	$F_{(6,109)}$= 2.41	p = **0.033**	$F_{(6,105)}$= 3.12	***p* = 0.008**	$F_{(6,107)}$= 1.57	p = 0.166	$F_{(3, 76)}$= 1.82	p = 0.154	$F_{(6,106)}$= 2.00	p = 0.075	$F_{(6, 104)}$= 2.15	***p* = 0.056**
**TE x HC**	$F_{(3,108)}$= 2.05	p = 0.113	$F_{(3,109)}$= 4.43	***p* = 0.006**	$F_{(3,105)}$= 1.29	p = 0.282	$F_{(3,107)}$= 1.20	p = 0.316	$F_{(3, 76)}$= 9.41	p< **0.001**	$F_{(3,106)}$= 2.99	***p* = 0.036**	$\mathrm{F}_{(3, 104)}$= 1.35	p = 0.265
**S x HC**	$F_{(2,108)}$= 24.28	***p*< 0.001**	$F_{(2,109)}$= 0.62	p = 0.542	$F_{(2,105)}$= 11.66	***p*< 0.001**	$F_{(2,107)}$= 0.55	p = 0.576	$F_{(1, 76)}$= 0.20	p = 0.655	$F_{(2,106)}$= 2.32	p = 0.104	$F_{(2, 104)}$= 5.15	***p* = 0.008**
**TE x S x HC**	$F_{(5,108)}$= 0.06	p = 0.997	$F_{(5,109)}$= 0.48	p = 0.793	$F_{(5,105)}$= 1.79	p = 0.124	$F_{(5,107)}$= 1.12	p = 0.357	$\mathrm{F}_{(3, 76)}$= 1.72	p = 0.172	$F_{(5,106)}$= 2.05	p = 0.080	$F_{(5,104)}$= 0.87	p = 0.507
