# Supplementary material for: Chronic environmental stress enhances tolerance to seasonal gradual warming in marine mussels
Source: PLoS One. 2017 Mar 23;12(3):e0174359. doi: 10.1371/journal.pone.0174359 (PMC5363927; doi:10.1371/journal.pone.0174359)
Supplement: S3 Table — (PDF) [file pone.0174359.s003.pdf]

S3 Table. Summary of Kruskall-Wallis[a] and one-way ANOVA[b] performed to analyze the effect of temperature elevation in studied biomarkers in healthy mussels (from Mundaka) and stressed mussels (from Arriluze) in fall, winter and summer. χ^2 : Chi square; *F*: Fisher's *F*; degrees of freedom are given between brackets; *p*: significance. Significant effects are indicated by bold characters (*p*<0.05).

	Healthy mussel population (Mundaka)						Stressed mussel population (Arriluze)					
	Fall		Winter		Summer		Fall		Winter		Summer	
**LP**[a]	$\chi^2_{(4)}$=17.84	***p*** **=0.001**	$\chi^2_{(3)}$=2.82	*p* =0.419	$\chi^2_{(4)}$=15.05	***p*** **<0.001**	$\chi^2_{(4)}$=2.52	*p* =0.641	$\chi^2_{(4)}$=9.27	*p* =0.055	$\chi^2_{(4)}$=10.00	***p*** **=0.040**
**Vv$_L$**[b]	$F_{(4,19)}$=3.89	***p*** **=0.018**	$F_{(3,9)}$=2.63	*p* =0.114	$F_{(3,10)}$=14.45	***p*** **=0.001**	$F_{(4,17)}$=0.58	*p* =0.680	$F_{(4,14)}$=1.28	*p* =0.329	$F_{(4,15)}$=0.41	*p* =0.797
**S/V$_L$**[a]	$\chi^2_{(4)}$=4.24	*p* =0.374	$\chi^2_{(3)}$=3.28	*p* =0.351	$\chi^2_{(3)}$=10.88	***p*** **=0.012**	$\chi^2_{(4)}$=2.69	*p* =0.610	$\chi^2_{(4)}$=1.19	*p* =0.880	$\chi^2_{(4)}$=2.82	*p* =0.588
**Nv$_L$**[a]	$\chi^2_{(4)}$=11.13	***p*** **=0.025**	$\chi^2_{(3)}$=6.01	*p* =0.111	$\chi^2_{(3)}$=4.36	*p* =0.225	$\chi^2_{(4)}$=13.32	***p*** **=0.010**	$\chi^2_{(4)}$=8.76	*p* =0.067	$\chi^2_{(4)}$=8.92	*p* =0.063
**Vv$_{NL}$**[a]	$\chi^2_{(4)}$=12.71	***p*** **=0.013**	$\chi^2_{(4)}$=5.52	*p* =0.238	$\chi^2_{(3)}$=6.32	*p* =0.097	$\chi^2_{(4)}$=11.26	***p*** **=0.024**	$\chi^2_{(4)}$=2.49	*p* =0.646	$\chi^2_{(4)}$=10.71	***p*** **=0.030**
**Vv$_{BAS}$**[b]	$F_{(4,18)}$=3.86	***p*** **=0.020**	$F_{(4,19)}$=2.59	*p* =0.070	$F_{(3,16)}$=8.07	***p*** **=0.002**	$F_{(4,18)}$=2.74	*p* =0.061	$F_{(4,19)}$=2.22	*p* =0.105	$F_{(4,19)}$=0.84	*p* =0.516
**MET**[b]	$F_{(4,18)}$=0.80	*p* =0.543	$F_{(4,19)}$=6.53	***p*** **=0.002**	$F_{(3,16)}$=0.80	*p* =0.510	$F_{(4,18)}$=1.45	*p* =0.259	$F_{(4,19)}$=1.23	*p* =0.332	$F_{(4,19)}$=1.69	*p* =0.193
**MLR**[b]	$F_{(4,18)}$=0.76	*p* =0.562	$F_{(4,19)}$=2.45	*p* =0.082	$F_{(3,16)}$=0.12	*p* =0.949	$F_{(4,18)}$=11.15	***p*** **<0.001**	$F_{(4,19)}$=4.86	***p*** **=0.007**	$F_{(4,19)}$=0.29	*p* =0.882
**MLR/MET**[b]	$F_{(4,18)}$=0.96	*p* =0.453	$F_{(4,19)}$=5.91	***p*** **=0.003**	$F_{(3,16)}$=0.37	*p* =0.773	$F_{(4,18)}$=7.82	***p*** **=0.001**	$F_{(4,19)}$=2.79	*p* =0.560	$F_{(4,19)}$=0.65	*p* =0.636
**CTD ratio**[b]	$F_{(4,18)}$=0.41	*p* =0.798	$F_{(4,19)}$=7.71	***p*** **=0.001**	$F_{(3,16)}$=1.12	*p* =0.37	$F_{(4,18)}$=0.93	*p* =0.470	$F_{(4,17)}$=1.53	*p* =0.237	$F_{(4,18)}$=2.76	*p* =0.059
**TAOC**[b]	$F_{(3,14)}$=4.32	***p*** **=0.024**	$F_{(3,15)}$=4.05	***p*** **=0.027**	$F_{(2,12)}$=0.96	*p* =0.412	$F_{(3,14)}$=2.44	***p*** **=0.180**	$F_{(3,15)}$=0.94	*p* =0.448	$F_{(3,15)}$=1.24	*p* =0.329
**PK**[b]	$F_{(3,15)}$=0.75	*p* =0.542	$F_{(3,15)}$=0.85	*p* =0.489	$F_{(2,11)}$=2.45	*p* =0.131	$F_{(3,15)}$=1.17	*p* =0.354	$F_{(3,14)}$=3.07	*p* =0.063	$F_{(3,16)}$=1.09	*p* =0.381
**PEPCK**[b]	$F_{(3,14)}$=3.65	***p*** **=0.039**	$F_{(3,14)}$=5.22	***p*** **=0.013**	$F_{(2,11)}$=2.91	*p* =0.097	$F_{(3,16)}$=1.74	*p* =0.200	$F_{(3,14)}$=3.1	*p* =0.061	$F_{(3,15)}$=12.48	***p*** **<0.001**
**PK/PEPCK**[b]	$F_{(3,15)}$=4.14	***p*** **=0.025**	$F_{(3,13)}$=7.05	***p*** **=0.005**	$F_{(2,10)}$=22.71	***p*** **<0.001**	$F_{(3,15)}$=1.15	*p* =0.362	$F_{(3,15)}$=1.01	*p* =0.415	$F_{(3,15)}$=2.87	*p* =0.071
**COX**[b]	$F_{(3,14)}$=1.90	*p* =0.177	$F_{(3,14)}$=0.26	*p* =0.853	$F_{(2,10)}$=3.30	*p* =0.079	$F_{(3,15)}$=3.30	*p* =0.059	$F_{(3,13)}$=0.82	*p* =0.506	$F_{(3,14)}$=3.89	***p*** **=0.032**
**HK**[b]	$F_{(3,14)}$=1.84	*p* =0.186	$F_{(3,15)}$=2.06	*p* =0.149	$F_{(2,11)}$=0.12	*p* =0.891	$F_{(3,15)}$=0.89	*p* =0.467	$F_{(3,13)}$=5.23	***p*** **=0.014**	$F_{(3,14)}$=4.46	***p*** **=0.021**
**GP**[b]	$F_{(3,12)}$=0.45	*p* =0.720	$F_{(3,14)}$=1.30	*p* =0.313	$F_{(2,10)}$=3.47	*p* =0.072	$F_{(3,14)}$=3.62	***p*** **=0.040**	$F_{(3,16)}$=0.04	*p* =0.991	$F_{(3,14)}$=2.74	*p* =0.083
