# Supplementary material for: Chronic environmental stress enhances tolerance to seasonal gradual warming in marine mussels
Source: PLoS One. 2017 Mar 23;12(3):e0174359. doi: 10.1371/journal.pone.0174359 (PMC5363927; doi:10.1371/journal.pone.0174359)
Supplement: S4 Table — (PDF) [file pone.0174359.s004.pdf]

S4A Table. Significant linear regressions of cell and tissue level biomarkers against experimental time after temperature elevation in fall, winter and summer for the cases of healthy mussels from Mundaka and stressed mussels from Arriluze

Healthy mussel population (Mundaka)			
**LP:**	$y_{(fall)}$=20.79-0.64x; R^2=0.62, *p*<0.001	$y_{(winter)}$=20.42+0.10x; R^2=0.04, *p*=0.53	$y_{(summer)}$=19.38-0.96x; R^2=0.41, *p*=0.03
**Vv_L:**	$y_{(fall)}$=0.005+0.0001x; R^2=0.30, *p*=0.006	$y_{(winter)}$=0.0005+4.01e^{-5}x; R^2=0.34, *p*=0.03	$y_{(summer)}$=0.0002+0.0002x; R^2=0.80, *p*<0.0001
**S/V_L:**	$y_{(fall)}$=2.74-0.005x; R^2=0.01, *p*=0.63	$y_{(winter)}$=6.75-0.086x; R^2=0.27, *p*=0.07	$y_{(summer)}$=8.6-0.76x+0.029x^2; R^2=0.99, *p*<0.0001
**Nv_L:**	$y_{(fall)}$=0.002+5.9e^{-5}x; R^2=0.25, *p*=0.01	$y_{(winter)}$=0.002+8.9e^{-5}x; R^2=0.45, *p*=0.01	$y_{(summer)}$=0.002+0.0001x; R^2=0.40, *p*=0.01
**Vv_{BAS}:**		$y_{(winter)}$=0.09+0.02x; R^2=0.24, *p*=0.02	
**MLR/MET:**	$y_{(fall)}$=0.93+0.007x; R^2=0.19, *p*=0.05	$y_{(winter)}$=0.95+0.018x; R^2=0.42, *p*=0.001	$y_{(summer)}$=1.06+0.002x; R^2=0.003, *p*=0.82
**CTD ratio:**	$y_{(fall)}$=0.305+0.008x; R^2=0.19, *p*=0.04	$y_{(winter)}$=0.21+0.007x; R^2=0.24, *p*=0.01	$y_{(summer)}$=0.234+0.004x; R^2=0.07, *p*=0.24
Stressed mussel population (Arriluze)			
**LP:**	$y_{(fall)}$=5.70+0.03x; R^2=0.03, *p*=0.41	$y_{(winter)}$=14.16-0.33x; R^2=0.72, *p*<0.001	$y_{(summer)}$=10.04-0.14x; R^2=0.27, *p*=0.04
**Nv_L:**	$y_{(fall)}$=0.002+4.7e^{-5}x; R^2=0.41, *p*=0.001	$y_{(winter)}$=0.0043+9.3e^{-5} x; R^2=0.12, *p*=0.16	$y_{(summer)}$=0.002+7.9e^{-5}x; R^2=0.49, *p*=0.001
**Vv_{BAS}:**	$y_{(fall)}$=0.115-0.002x; R^2=0.23, *p*=0.02		
**MLR/MET:**	$y_{(fall)}$=0.69-0.008x; R^2=0.18, *p*=0.04		
**CTD ratio:**	$y_{(fall)}$=0.64-0.011x; R^2=0.19, *p*=0.04	$y_{(winter)}$=0.303+0.008x; R^2=0.26, *p*=0.015	$y_{(summer)}$=0.28+0.003x; R^2=0.10, *p*=0.14

S4B Table. Statistical significance (*p*<0.05) of the linear regression coefficients of cell and tissue level biomarkers against experimental time after temperature elevation in fall, winter and summer. Significant effects are indicated by bold characters (*p*<0.05).

	Healthy mussel population (Mundaka)			Stressed musel population (Arriluze)		
	**Fall**	**Winter**	**Summer**	**Fall**	**Winter**	**Summer**
**LP**	t(β_1)= -6.152 *p*< **0.001**	t(β_1)= 0.650 *p* = 0.532	t(β_1)= -2.514 *p* = **0.033**	t(β_1)= 0.846 *p* = 0.407	t(β_1)= -5.842 *p*< **0.001**	t(β_1)= -2.290 *p* = **0.038**
**Vv_L**	t(β_1)= 3.045 *p* = **0.006**	t(β_1)= 2.405 *p* = **0.035**	t(β_1)= 6.880 *p*< **0.001**			
**S/V_L**	t(β_1)= 3.218 *p* = **0.004**	t(β_1)= 2.811 *p* = **0.017**	t(β_1)= -5.268 *p*< **0.001**			
			t(β_2)= 3.223 *p* = **0.008**			
**Nv_L**	t(β_1)= 2.718 *p* = **0.013**	t(β_1)= 3.032 *p* = **0.011**	t(β_1)= 2.835 *p* = **0.015**	t(β_1)= 3.754 *p* = **0.001**	t(β_1)= 1.479 *p* = 0.159	t(β_1)= 4.141 *p* = **0.001**
**Vv_{BAS}**		t(β_1)= 2.610 *p* = **0.016**		t(β_1)= -2.479 *p* = **0.022**		
**MLR/MET**	t(β_1)= 2.081 *p* = **0.050**	t(β_1)= 4.010 *p* = **0.001**	t(β_1)= 0.225 *p* = 0.824	t(β_1)= 2.163 *p* = **0.042**		
**CTD ratio**	t(β_1)= 2.175 *p* = **0.042**	t(β_1)= 2.673 *p* = **0.014**	t(β_1)= 1.205 *p* = 0.244	t(β_1)= -2.167 *p* = **0.042**	t(β_1)= 2.658 *p* = **0.015**	t(β_1)= 1.544 *p* = 0.138
